# Autofluorescence as a measure of senescence in *C. elegans*: look to red, not blue or green

**DOI:** 10.18632/aging.100936

**Published:** 2016-04-10

**Authors:** Zachary Pincus, Travis C. Mazer, Frank J. Slack

**Affiliations:** ^1^ Department of Developmental Biology, Washington University School of Medicine, St. Louis, MO 63110, USA; ^2^ Department of Genetics, Washington University School of Medicine, St. Louis, MO 63110, USA; ^3^ Institute for RNA Medicine, Department of Pathology, Beth Israel Deaconess Medical Center, Harvard Medical School, Boston, MA 02215, USA

**Keywords:** autofluorescence, lipofuscin, age pigment, health

## Abstract

In *C. elegans*, intestinal autofluorescence (sometimes referred to as lipofuscin or “age pigment”) accumulates with age and is often used as a marker of health or the rate of aging. We show that this autofluorescent material is spectrally heterogeneous, and that materials that fluoresce under different excitation wavelengths have distinct biological properties. Red autofluorescence (visible with a TRITC filterset) correlates well with an individual's remaining days of life, and is therefore a candidate marker of health. In contrast, blue autofluorescence (via a DAPI filterset) is chiefly an indicator of an individual's incipient or recent demise. Thus, population averages of blue fluorescence essentially measure the fraction of dead or near-dead individuals. This is related to but distinct from the health of the living population. Green autofluorescence (via a FITC or GFP filterset) combines both properties, and is therefore ill suited as a marker of either death or health. Moreover, our results show that care must be taken to distinguish GFP expression near the time of death from full-body green autofluorescence. Finally, none of this autofluorescence increases after oxidative stress, suggesting that the material, or its biology in *C. elegans*, is distinct from lipofuscin as reported in the mammalian literature.

## INTRODUCTION

Autofluorescent material builds up over time in cells and tissues with low turnover, and is often used as a marker of cellular and even organismal aging [1]. This material, first described almost 175 years ago [2], is generically described as “age pigment” or lipofuscin, and has been characterized extensively in many different organisms [3–9]. Viewed *in vivo*, lipofuscin typically fluoresces in yellow to red wavelengths when excited with UV or blue light [10], and is believed to consist of highly-oxidized, insoluble cross-linked proteins and lipids [8,11]. However, the exact spectral properties, and likely the precise chemical nature, of this material varies across tissues and organisms [8]. The material generally increases with age and with oxidative damage [7]; related materials described as “ceroid” have been shown to increase with specific disease processes as well [8,11].

In *Caenorhabditis elegans*, where the entire soma is post-mitotic, autofluorescence has long been noted as a correlate of aging [12–14]. In *C. elegans*, as in mammalian cells, much of the autofluorescence is confined to intracellular granules of lysosomal origin, which, in *C. elegans*, are generally found within intestinal cells [15]. The precise relationship between autofluorescence, aging, and lifespan in *C. elegans* has remained somewhat ill defined, however. In particular, different groups have come to different conclusions regarding whether autofluorescence in particular wavelengths does [16] or does not [17] increase with age as a reflection of the overall health of a population of animals, and whether autofluorescence in a particular individual does [16,18] or does not [19] correlate with its health and/or future lifespan.

**Figure 1:**
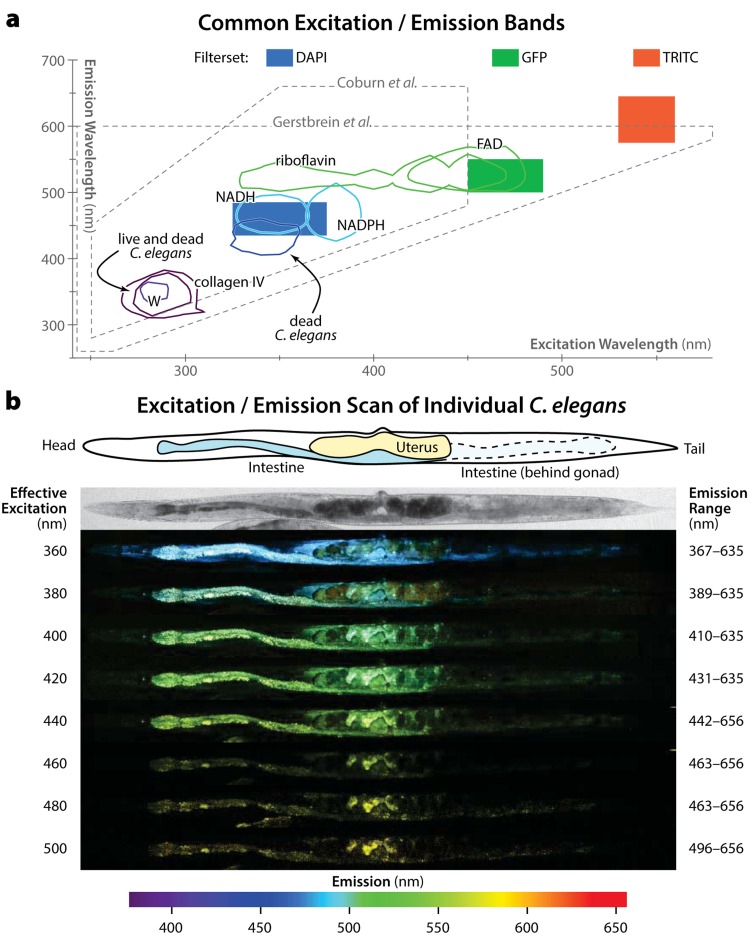
Spectral properties of autofluorescent materials in *C. elegans* **(a)** Fluorescent excitation/emission wavelengths of note are show. First, using data from DaCosta *et al*. [28], we plot contours containing 75% of the overall fluorescence emission intensity for several common autofluorescent biomolecules: tryptophan (W), collagen IV, NADH, NADPH, riboflavin, and FAD. Next, we plot the 75% contours of autofluorescent peaks for living and dead *C. elegans* using data from Coburn *et al*. [17]. The excitation/emission range analyzed in that work is shown with a dashed line. The range analyzed by a previous quantitative study of *C. elegans* autofluorescence by Gerstbrein *et al*. [16] is likewise shown. Of note, neither study covered excitation and emissions in the yellow/red wavelengths. The data presented in this work was generated using three fluorescent filter sets with excitation/emission wavelengths as diagrammed by the colored boxes. Blue (DAPI filterset): 350/50 nm (center wavelength / bandwidth) excitation 460/50 nm emission; Green (GFP filterset): 470/40 nm ex, 525/50 nm em; Red (TRITC filterset): 545/30 nm ex, 610/70 nm em. **(b)** A single animal at the fourth day of adulthood (near the end of the reproductive span) was imaged with two-photon confocal microscopy in multiple excitation wavelengths using a tunable laser. For each effective excitation wavelength (i.e. the single-photon equivalent of half the actual laser wavelength), emissions in the specified wavelengths are shown. After computationally straightening the images, fluorescence emissions (captured into bins of ∼10 nm width) were false-colored according to their wavelength, per the color bar below. All emissions for a given excitation wavelength are shown superimposed into a single image. At top, the position of the intestine and uterus is sketched and shown in a brightfield image. At the rear of the animal, the intestine is overlapped by the posterior gonad arm, causing it to be slightly obscured (sketched by dashed lines). The uterus is filled with unfertilized oocytes, visible in the images as dark circles. Note that relative emission intensities can be compared within, but not between, specific excitation wavelengths.

Despite this, many studies use accumulation of intestinal autofluorescence in *C. elegans* to measure whether particular interventions improve health or slow aging. Moreover, there is little standardization regarding what excitation / emission wavelengths to use for this analysis. Some studies have focused on autofluorescence in blue emission wavelengths [13,16,17], others in cyan [20] and green ranges [19,21], and yet others on red emissions [18]. Figure 1a provides a schematic relating excitation and emission wavelengths of common fluorescent biomolecules to visible colors. This diversity likely reflects a discrepancy long noted in studies of lipofuscin and related compounds: viewed *in vivo*, these materials are generally observed to fluoresce with emission colors from yellow-green to red; however, when solvent-extracted for detailed characterization, the same materials fluoresce in blue wavelengths [10,22]. *C. elegans* studies with their roots in the biochemical characterization of these materials [12,14] typically focus on UV excitation with blue emissions, while other studies focus on the green-to-red color familiar to *C. elegans* microscopists.

Last, recent work using data from both *in vivo* studies and in solvent extracts has shown that several hours before and after death, individuals become dramatically more autofluorescent in blue wavelengths (excitation/emission wavelengths centered on 340/430 nm; Figure 1a) [17]. Moreover, autofluorescence in these wavelengths appears to be specific to dead and dying individuals. This leads to the possibility that increases in blue autofluorescence over time observed in populations of aging *C. elegans* may reflect not the aging rate or health state of the population *per se*, but instead the fraction of dead or almost-dead individuals in the sample. Though obviously related, these two measures are distinct.

In order to reconcile these discordant findings, we examined autofluorescence in three common excitation/emission bands (Figure 1a), following a population of identifiable individual *C. elegans* in longitudinal fashion from young adulthood until death. Because each individual was individually housed and its time of death manually annotated [18], fluorescence increases during aging can be distinguished from those that occur at the time of death only. Overall, we find that autofluorescent material in *C. elegans* is spectrally and biologically more complex than previously understood. We confirm that “blue” autofluorescence (ex/em 350/460 nm) increases very little across aging except for a peak near death. In contrast, “red” autofluorescence (ex/em 546/600 nm) increases linearly over time, and is well correlated with each individual's future lifespan (a proxy for health). Last, “green” autofluorescence (ex/em 470/525 nm) combines both characteristics. Finally, we confirm that increasing oxidative damage (via treatment with redox-active iron) does not increase accumulation of blue autofluorescent materials [17]; we further find that such treatment does not increase green or red autofluorescence either. This is in clear contrast to the literature on lipofuscin in mammalian systems, in which autofluorescence has consistently been reported to increase subsequent to oxidative damage generally, and iron treatment specifically. Thus, none of the materials in the different spectral bands that we studied behave similarly to lipofuscin as reported in the mammalian literature. As such, this work does not refer to any autofluorescence in *C. elegans* as “lipofuscin”. We further remain neutral as to the chemical makeup of the different fluorescent species described here.

## RESULTS

### Autofluorescence in C. elegans is spatially and spectrally complex

We first sought to determine whether autofluorescence in *C. elegans* is due to a homogenous material. To qualitatively characterize this material, we performed an excitation/emission scan of a live, aged *C. elegans* using confocal microscopy with a tunable laser for excitation and a detector capable of resolving emission spectra at each image pixel. The results, shown in Figure 1b, demonstrate that there are two main sources of autofluorescence in *C. elegans*: the intestine and the uterus. In animals of this age the uterus is often filled with unfertilized oocytes containing yolk proteins, which are known to be autofluorescent. Therefore we speculate that some of the uterine fluorescence observed may be due to yolk accumulation. Regardless, the autofluorescence in this organ is distinctly heterogeneous in terms of emission wavelengths from any given excitation, and is distinct from the autofluorescence of the intestine. The material in the intestine is also qualitatively not homogenous: for any given excitation, the material fluoresces with more blue-shifted or more yellow-shifted emissions in different spatial regions. These differences are more subtle in the intestine than uterus, however.

### Red autofluorescence increases linearly with time, blue peaks near death only

Overall, autofluorescence in *C. elegans* appears to be due to a spatially heterogeneous mix of substances with distinct spectral properties. We next sought to determine how this autofluorescence changes in time. To do so, we used a culture apparatus in which each individual animals are isolated and can be imaged under a widefield fluorescent microscope *in situ* without having to be transferred to a microscope slide [18]. In this way, we were able to collect longitudinal timecourses of autofluorescence for 40 individual animals from hatching to death in a minimally invasive fashion.

Images at 10× magnification in the three fluorescence ranges illustrated in Figure 1a were collected for each individual every eight hours. Animals were assumed to be alive after each time-point if there was visible movement following blue-light stimulus. This produced a detailed survey of *C. elegans* autofluorescence across space and time, and among individuals.

**Figure 2:**
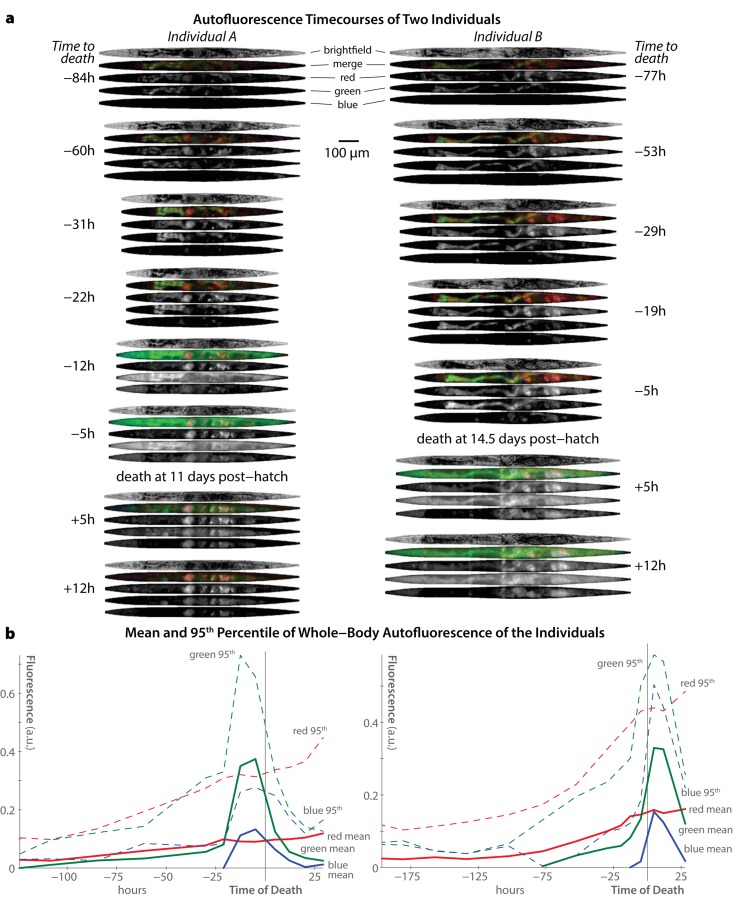
Trends in autofluorescence over time in individual *C. elegans* **(a)** Computationally straightened brightfield and fluorescence images of two different individuals (left and right) at selected time-points are shown. The timecourse for each individual is shown top-to-bottom, with images annotate by the number of hours before/after that individual's death. Fluorescence images obtained in the “red”, “green”, and “blue” channels (see Figure 1) are shown individually and in false-color merge images for each time-point. Note the peak in green and blue fluorescence near each individual's time of death (as ascertained by cessation of movement following stimulation). **(b)** Whole-body fluorescence across the entire experiment is shown for the two individuals pictured in panel a. For each fluorescent channel, the mean and 95^th^ percentile intensity of all image pixels in the animal's body is plotted over time.

Figure 2 shows selected images throughout the life of two specific individuals from our study. Several major trends are clear. First, increases in blue autofluorescence are almost exclusively a near-death phenomenon, appearing from 6–12 hours before each individual's death. As previously described, this autofluorescence is predominantly intestinal in nature [17]. While we observe some blue autofluorescence in the gonad and uterus, this material is co-localized with red autofluourescence in that organ, and does not appear to increase near death. Green autofluorescent material, in contrast, is observable throughout life, primarily in the intestine. Near death, green autofluorescence increases dramatically in the intestine and also peripheral tissues. Finally, red autofluorescent material is observable in both the intestine and gonad/uterus throughout life, and does not noticeably increase near death. Interestingly, though both red and green materials are visible in the intestine, there is limited spatial overlap within this tissue. This suggests that these materials are biologically distinct from one another, in agreement with the observations of spatial inhomogeneity in autofluorescence from the spectral imaging shown in Figure 1.

Figure 2b demonstrates that temporal trends in autofluorescence are similar regardless of whether the mean whole-body fluorescent intensity is (solid lines) or 95^th^-percentile intensity (dashed lines) is plotted. The 95^th^-percentile intensity, calculated across all body pixels, is an outlier-resistant proxy for maximum fluorescence intensity. According to both measures, blue autofluorescence remains static until a dramatic peak near death, red autofluorescence increases roughly linearly with time, and green autofluorescence appears to combine both trends.

### Population averages of blue autofluorescence are confounded by dead and dying individuals

**Figure 3:**
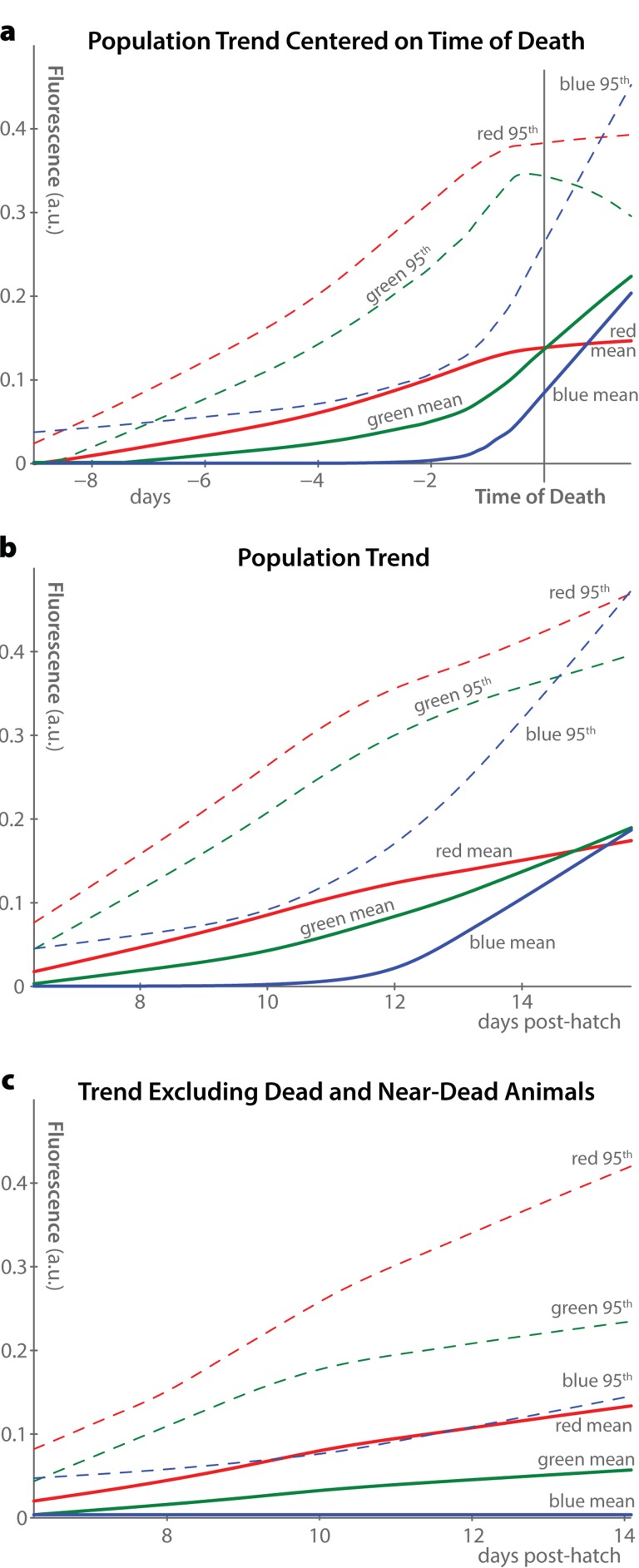
Population trends in autofluorescence **(a)** Average trends in autofluorescence across all 40 individuals in the dataset are plotted, aligned at the time of death of each individual. Two measurements of fluorescence were calculated for each individual at each timepoint in each fluorescence channel: the mean whole-body fluorescence intensity, and the 95^th^ percentile of fluorescence intensity. LOWESS nonparametric regression was used to fit smooth curves through the measured data, producing the shown population-average trends. **(b)** Curves similar to panel a are plotted, using the time of hatching rather than time of death as the alignment point. **(c)** By excluding animals within 12 hours of death from the plots in panel b, it becomes clear that the majority of the increase in blue and green fluorescence, but not red fluorescence, is driven by increases immediately before organismal death.

While Figure 2 presents data from only two individuals, the same measures averaged across the 40-animal population are shown in Figure 3. Mean and 95^th^-percentile fluorescence were calculated for each individual at each time-point, and the mean of these measures across the whole population is plotted as a function of time. This analysis shows that, when centered at the time of death (Figure 3a), the population trends are very similar to the individual trends described above: a consistent increase of red autofluorescence over time, a peak in blue autofluorescence only near death, and a combination of both of these patterns in green.

Without individual-level longitudinal analysis, however, it is not possible to recenter time trends around each individual's death. As such, population-average fluorescence measurements usually appear as in Figure 3b, where average autofluorescent trends are plotted against time of hatching. In this analysis, blue autofluorescence appears to increase somewhat over time in the population. However, this effect is driven almost entirely by dead and near-dead animals, as shown in Figure 3c, which excludes animals within 12 hours of death from the trend. Therefore, we suspect that previous observations of increasing blue autofluorescence in aging *C. elegans* were effectively measures of the fraction of dead and dying animals in the study populations.

### Red autofluorescence is the best predictor of future lifespan in *C. elegans*

As discussed above, previous studies have used blue autofluorescence to compare aging, aging rates, and health between different populations of *C. elegans.* In general, longer-lived populations have been found to have lower blue autofluorescence at any given time-point. As before, our results suggest that this simply reflects a smaller proportion of near-dead animals, rather than a slowed aging rate *per se*. (These two parameters are obviously quite related, however. Thus, results of analyses of blue fluorescence remain meaningful, though their precise interpretation must be revised.)

In order to shed light on which autofluorescent wavelengths are most reflective of individual and population senescence, we asked which measures best correlate with an individual's remaining lifespan. While “senescence” is a difficult concept to define, it is clear that among animals of the same age, those with more days to live are in a meaningful sense less senescent than those with fewer days remaining. Therefore we adopt this measure as a plausible, though certainly imperfect, proxy for senescence.

**Figure 4:**
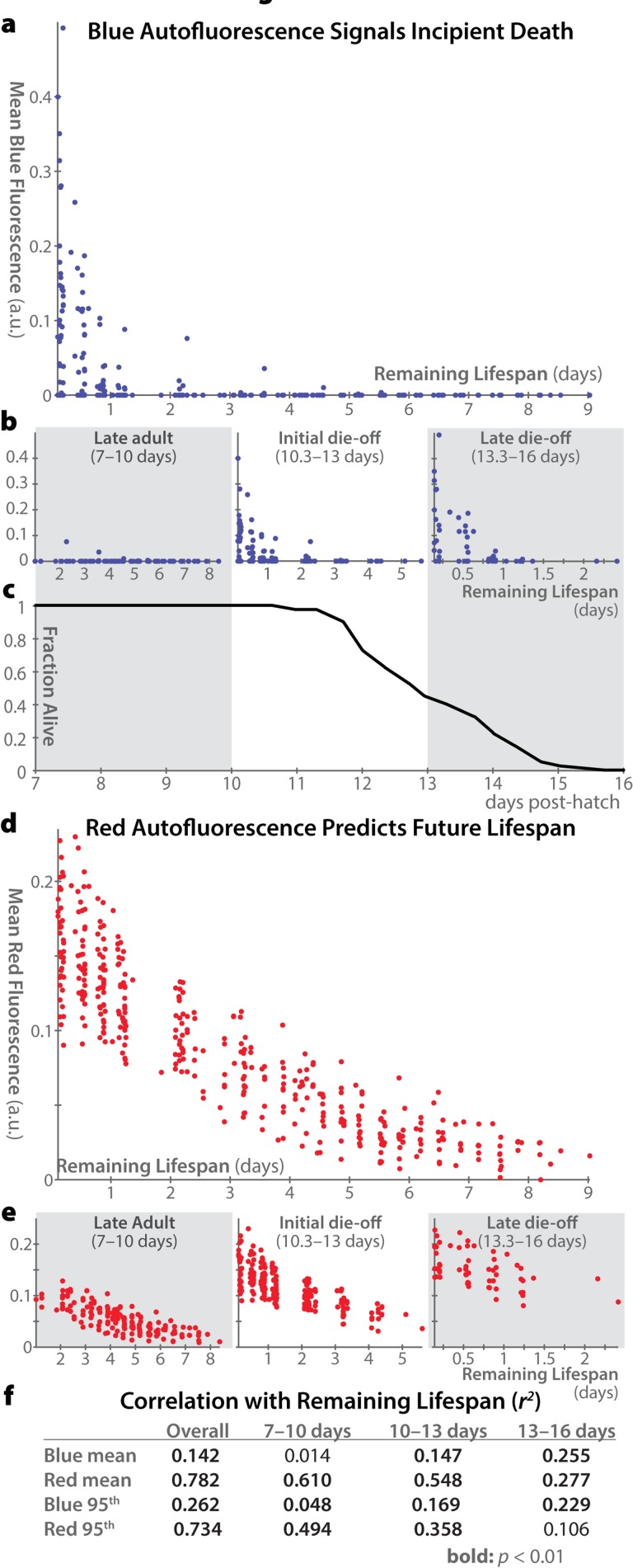
Correlations between autofluorescence and remaining lifespan **(a)** Whole-body mean blue autofluorescence is plotted for each individual at each timepoint versus that individual's remaining lifespan at that timepoint (a rough proxy for individual health). **(b)** Plots as in panel a are shown for animals in different age ranges: 7–10 days post hatch (in which range all of the population remains alive), 10.3–13 days (during which approximately the first half of the deaths occur), and 13.3–16 days (during which the remaining deaths occur). Correlation between blue autofluorescence and future lifespan is most pronounced only in older animals with very few days of life remaining. **(c)** Lifespan curve for the 40 individuals in the experiment showing the fraction of animals alive within each of the time ranges plotted in panel b. **(d, e)** Mean red autofluorescence versus remaining lifespan is plotted for all individuals at all timepoints (d) and for specific age ranges (e), as in panels a and b. **(f)** Pearson coefficients of determination (*r^2^*) between autofluorescence and remaining lifespan are shown for whole-body mean and 95^th^-percentile fluorescence in different age ranges. Values with *p-*values < 0.01 according to an F-test are shown in bold. Overall, whole-body mean red autofluorescence can account for 78.2% of the total variability in lifespan remaining in a population of adult animals.

Figure 4 shows remaining lifespan plotted against autofluorescence for each individual in our population, at each time-point measured. Across all ages, blue autofluorescence is weakly correlated with remaining lifespan, and this correlation is driven entirely by animals within a day of death (Figure 4a). Moreover, for animals in late adulthood (after the reproductive span but before the onset of mortality), blue autofluorescence is entirely unrelated to remaining lifespan (Figure 4b). In contrast, red autofluorescence is highly predictive of remaining lifespan in the overall population, in late adulthood, and in older populations (Figure 4d,e). Only in highly aged animals in which nearly the whole population is within a day of demise does the level of blue autofluorescence become more predictive of remaining lifespan than that of red autofluorescence.

These results confirm our previous findings [18] that the 95^th^-percentile of whole-body red autofluorescence is well correlated with an individual's future lifespan. The difference in future lifespan between individuals with high vs. low levels of red autofluorescence is quite pronounced. This difference peaks at day 10 post-hatch (a time when <5% of the population has died). At this age, individuals with lower-than-average red autofluorescence go on to live an average of 1.66 days longer than those with higher-than-average red autofluorescence. (For scale, the standard deviation of lifespans in our dataset is 1.4 days.) From days 7–12 post-hatch (inclusive), the difference in mean remaining between brightly autofluorescent and dimly autofluorescent animals is over 1 day.

Based on this new analysis, red autofluorescence (mean or 95^th^ percentile) is the best proxy measure for individual and population health. Blue auto-fluorescence, in contrast, predicts essentially only incipient demise. As green autofluorescence appears to combine properties of both blue and red auto-fluorescence (increase over time as well as a peak near death), we believe it is difficult to interpret fluorescence levels in these wavelengths, and did not include it in this analysis. Moreover, green autofluorescence has been previously reported to not correlate with future lifespan [19].

### Oxidative damage does not increase auto-fluorescence in *C. elegans*

**Figure 5:**
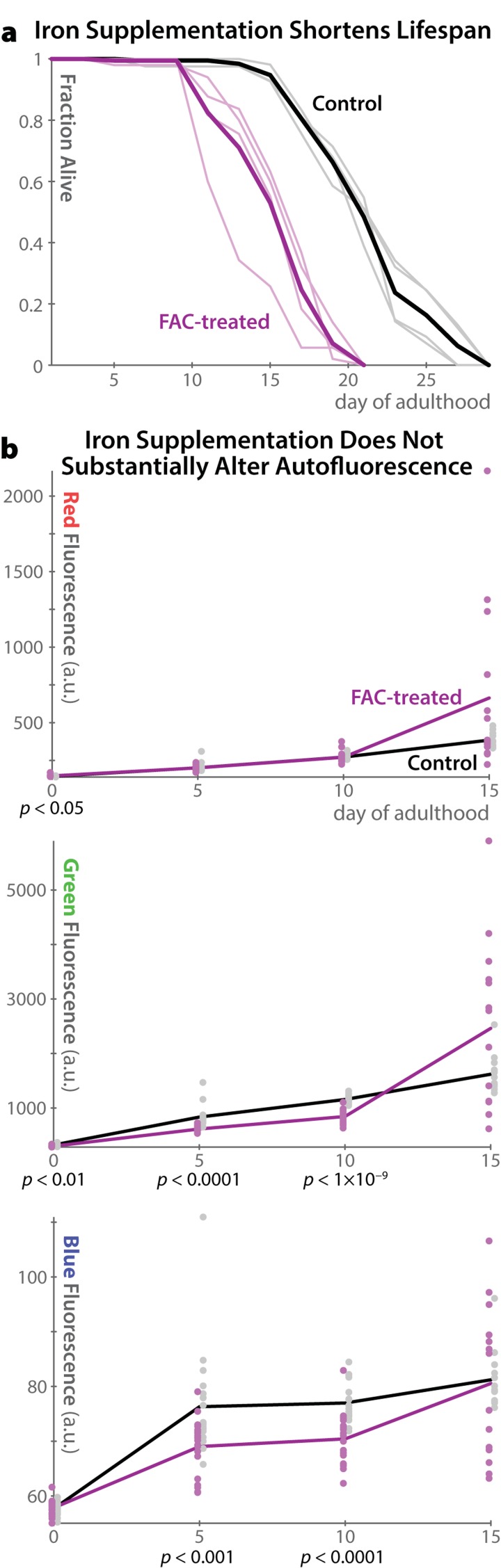
Autofluorescence in *C. elegans* does not increase in pro-oxidation conditions **(a)** Supplementation of the media with Fe(III) in the form of 15 mM ferric ammonium citrate (FAC) shortens lifespan as previously reported [23]. Four replicate plates of 50 individuals each were analyzed for FAC and control conditions (light lines). The heavy line shows the overall average for each condition. **(b)** Despite shortening lifespan, iron supplementation does not increase autofluorescence levels in any channel investigated. While at several time-points *t-*test *p-*values indicate significant differences between the groups, the changes are relatively small in magnitude and are in all cases in the direction of *less* auto-fluorescence in the FAC group. Population variability is, however, somewhat increased at the last timepoint in the FAC group.

As lipofuscin is believed to consist of highly oxidized proteins and lipids, it is unsurprising that previous observations of mammals and mammalian cells have found that conditions in which oxidative damage is increased also raise lipofuscin levels. In particular, both high O_2_ concentrations and supplementation with redox-active iron increase endogenous autofluorescence in multiple *in vivo* and *in vitro* experiments in different mammalian models(reviewed in ref. [7]). In *C. elegans*, media supplemented with 15 mM ferric ammonium citrate has been shown to increase oxidative damage (as measured by protein carbonylation) [17,23] and shorten lifespan [23]. However, such supplementation was previously reported to not increase blue auto-fluorescence [17]. As mammalian lipofuscin is often reported to fluoresce in green to red wavelengths when viewed *in vivo* [10], we hypothesized that, though blue autofluorescence in *C. elegans* appears to be distinct from mammalian lipofuscin [17], green or red autofluorescence might increase following oxidative damage. We therefore repeated the analysis of *C. elegans* autofluorescence with redox-active iron supplementation from ref. 17, and extended it to green and red fluorescent wavelengths (Figure 5). We confirmed that iron supplementation shortens lifespan dramatically (Figure 5a); however autofluorescence is not much altered at any time in any wavelength (Figure 5b). Indeed, all significant changes between control and treatment groups are in the direction of *reduced* auto-fluorescence. Examining autofluorescence specifically in the intestine or gonad produced identical results (data not shown). While inter-individual variability in autofluorescence is increased in iron-treated animals at very late timepoints, there is no evidence of wholesale increases in blue, green, or red autofluorescence as a result of these pro-oxidation conditions. These results corroborate previous observation of no increase in blue autofluorescence in mutant *C. elegans* strains that produce high levels of reactive oxygen species [16], and stand in marked contrast to the behavior of lipofuscin as characterized in mammals.

## DISCUSSION

*C. elegans* autofluorescence is produced by a spectrally complex mixture of materials. Each of these autofluorescent materials accumulates to different degrees in different tissues, has distinct behaviors across time, and reflects distinct aspects of organismal physiology. Moreover, this autofluorescence does not respond to oxidative damage in the same way as the material known as lipofuscin from the mammalian literature. We therefore do not describe this material as such, and prefer to characterize the different autofluorescent materials in *C. elegans* spectrally, as “red”, “green”, or “blue” autofluorescence.

Jointly analyzing red, green, and blue autofluorescence together validates previous reports that blue autofluorescence does not substantially increase in *C. elegans* except at the very end of life [17], while red autofluorescence increases linearly over time in a manner highly predictive of future lifespan [18]. Overall, this work demonstrates the unsuitability of blue and green autofluorescence as a microscopy-based measure of population or individual health or aging. Instead, measures of blue autofluorescence are best considered as an estimate of the relative fraction of the population that is dead or nearly so at any time. In contrast, red autofluorescence increases monotonically and almost linearly with time, and is highly correlated with each individual's future lifespan. As such, it represents a potential proxy measure for health of individuals and between populations. Because green autofluorescence shares properties of both the blue and red materials, any measure of autofluorescence in green wavelengths is difficult to interpret.

The previous work of Gerstbrein *et al*. examined autofluorescence in populations of intact *C. elegans,* with spectrometric detail previously only available in solvent-extracted samples [16]. This careful study identified several trends in blue autofluorescence that are not readily explicable with a simple “blue autofluorescence only reflects death” model. In particular, these authors noted a spectral shift in blue autofluorescence of dietary-restricted vs. *ad libitum*-fed animals, which is not explicable by the different lifespans of these populations. We also note that even in our data, peak whole-body blue autofluorescence (measured as the 95^th^ percentile of pixel intensity across the animal) increases slightly but consistently with time (Figures 2 and 3). Therefore, there are clearly processes relevant to aging and health that produce some degree of blue autofluorescent material over time, independent of incipient death. While spectrometric equipment as deployed Gerstbrein *et al*. may be able to measure these processes, we caution that non-death increases in blue autofluorescence are very difficult to distinguish from background autofluorescence in widefield and even confocal imaging.

Our results also show that at organismal death, bright green autofluorescence appears diffusely throughout the entire body of *C. elegans*. This green signal is spectrally and spatially distinct from the previously reported blue intestinal autofluorescence at death [17], though its origin and chemical makeup may be very similar. Therefore, we urge extreme caution in interpreting measures of GFP in very old transgenic animals, especially when the “GFP” appears as a diffuse, whole-body fluorescent signal. In these cases, caution should be taken to ensure that there is no concomitant blue “death fluorescence” signal before assuming that the green fluorescent signal originates from GFP. In particular, note that Figure 2 shows that this green signal is largely un-accompanied by red autofluorescence. Therefore, unlike the traditional green-yellow intestinal autofluorescence well known to *C. elegans* microscopists, this near-death green signal appears as more “pure” green, and thus may be more easily confused with a true GFP signal.

In sum, autofluorescence in red wavelengths (as visualized by common TRITC/RFP filtersets) is most appropriate to use to characterize aging in *C. elegans*. Blue autofluorescence (via DAPI filtersets) is an excellent measure of the proportion of near-dead animals in a population. Green autofluorescence (via a GFP filterset) is difficult to interpret, and presents a danger to unwary investigators as strong near-death green autofluorescence may easily be confused with increases in GFP in transgenic animals.

## MATERIALS AND METHODS

### Spectral imaging

Seven day-post-hatch individuals (4^th^ day of adulthood at a culture temperature of 23.5°C) were mounted to a 2% agarose pad on a glass slide and immobilized under a coverslip with 10 mM levamisole. The animals were imaged on a Zeiss LSM 510 Meta confocal microscope using a tunable laser (Chameleon, Coherent Inc.) as an excitation source. The instrument was configured for two-photon excitation and the Meta monochromator was used to collect emission wavelengths in ∼10-nm bins. The resulting spectral images were computationally straightened as described previously [18]. For each excitation wavelength, the image of emissions from each wavelength bin was false-colored according to the wavelength using Bruton's algorithm, and combined using the screen image compositing operator.

### Individual time-course imaging and quantification

Using culture techniques previously described [18], we reared 40 individual animals on gel pads embedded in a glass slide, such that each individual was trapped on the gel surface between the pad and a thin layer of PDMS silicone acting as a flexible, gas-permeable, and optically transparent “coverslip”. These individuals, bearing the *spe-9(hc88)* temperature-sensitive sterility mutation (resulting in otherwise normal lifespans and physiology [24,25]), were deposited on the pads as embryos and reared at the restrictive temperature of 23.5°C. Every eight hours, the slides were placed on a Zeiss Axioskop microscope, each animal was manually located and brought into focus under 10× magnification, and fluorescence images were obtained using DAPI, GFP, and TRITC filtersets (Chroma Technology; filtersets 31000v2, 41017, and 41002c, respectively). At each time, images of autofluorescent beads (Molecular Probes) were also obtained to control for temporal variability in light-source intensity. Similarly, flat-field images using uniformly autofluorescent acrylic slides (Chroma Technology) were collected to control for spatial variability in fluorescence illumination. Fluorescent images were dark-current and flat-field corrected and divided by the average per-bead fluorescence, in order to produce fluorescence values comparable across space and between time-points. The same exposure time was used for each fluorescence channel across the full experiment.

Images of each individual were computationally straightened using custom software [18] and times of death were manually annotated. Autofluorescence intensities were quantified as follows. First, background autofluorescence due to the culture medium was estimated by calculating median fluorescence in a region adjacent to the position of the animal in each image. After subtracting this background value, the mean and 95^th^ percentile of intensity in the image pixels belonging to the animal's body were calculated and recorded. (Our findings are not substantially altered by omitting this background subtraction; data not shown.)

### Iron supplementation and imaging

15 mM ferric ammonium citrate (FAC) plates were prepared as previously described [23], and supplemented with 5-fluoro-2′-deoxyuridine (FUDR) to a final concentration of 40 μM to prevent reproduction [26,27]. Gravid wild-type (N2) *C. elegans* were hypochlorite treated to isolate age-synchronized first-larval stage offspring, which were reared on standard plates until the 4^th^ larval stage at which point individuals were transferred to FAC/FUDR plates and FUDR-only control plates. For lifespan assays, 50 individuals were transferred to each of four FAC and four control plates. For image acquisition, four additional FAC and control plates with 50 individuals each were prepared. At 0, 5, 10, and 15 days post transfer to FAC, individuals were mounted microscope slides with agarose pads and levamisole as above, and fluorescence images obtained. At days 0 and 5, 20 individuals were measured for each group; at day 10, 20 FAC and 19 control animals were examined; at day 15, 14 FAC and 13 control animals were examined.
